# Coronaviral Infection and Interferon Response: The Virus-Host Arms Race and COVID-19

**DOI:** 10.3390/v14071349

**Published:** 2022-06-21

**Authors:** Qi Liu, Sensen Chi, Kostyantyn Dmytruk, Olena Dmytruk, Shuai Tan

**Affiliations:** 1 Department of Immunology, School of Basic Medicine, Chongqing Medical University, Chongqing 400010, China; chisensen@stu.cqmu.edu.cn; 2 Department of Internal Medicine, University of Texas Southwestern Medical Center, Dallas, TX 75390, USA; 3 Department of Molecular Genetics and Biotechnology, Institute of Cell Biology, National Academy of Sciences of Ukraine, 79005 Lviv, Ukraine; dmytruk77@gmail.com (K.D.); verbaolena@gmail.com (O.D.); 4 Institute of Biology and Biotechnology, University of Rzeszow, 35-601 Rzeszow, Poland

**Keywords:** SARS-CoV-2, COVID-19, pattern-recognition receptor, PRR, interferon, innate immune, antiviral drug

## Abstract

The recent pandemic caused by severe acute respiratory syndrome coronavirus 2 (SARS-CoV-2) has resulted in unprecedented morbidity and mortality worldwide. The host cells use a number of pattern recognition receptors (PRRs) for early detection of coronavirus infection, and timely interferon secretion is highly effective against SARS-CoV-2 infection. However, the virus has developed many strategies to delay interferon secretion and disarm cellular defense by intervening in interferon-associated signaling pathways on multiple levels. As a result, some COVID-19 patients suffered dramatic susceptibility to SARS-CoV-2 infection, while another part of the population showed only mild or no symptoms. One hypothesis suggests that functional differences in innate immune integrity could be the key to such variability. This review tries to decipher possible interactions between SARS-CoV-2 proteins and human antiviral interferon sensors. We found that SARS-CoV-2 actively interacts with PRR sensors and antiviral pathways by avoiding interferon suppression, which could result in severe COVID-19 pathogenesis. Finally, we summarize data on available antiviral pharmaceutical options that have shown potential to reduce COVID-19 morbidity and mortality in recent clinical trials.

## 1. Introduction

SARS-CoV-2, the pathogen that causes development of coronavirus disease 2019 (COVID-19), was identified on 7 December 2019 [1] in China and quickly spread worldwide. Based on the data from the World Health Organization, there are more than 530 million confirmed cases of COVID-19, with the global death toll exceeding 6.3 million deaths [2]. However, physicians quickly learned that children are typically protected from the severe COVID-19 disease. That was surprising because young children are typically the most vulnerable population together with older adults for most viruses. Further research highlighted a possible role of innate immunity in SARS-CoV-2 suppression, which could explain children’s resistance to COVID-19 (due to trained innate immunity), as well as vulnerability of older adult and immunocompromised patients who cannot properly suppress viral replication in first days of infection. Here, we collect recent data, suggesting that the interferon system is an essential arm that cells engage against SARS-CoV-2. At the same time, the virus tries to avoid its suppression through a number of evasion weapons.

## 2. Overview of the SARS-CoV-2 Life Cycle

SARS-CoV-2 belongs to the Coronaviridea family, which consists of enveloped single-stranded RNA viruses. The family can be divided into two subfamilies, the Coronavirinae and the Torovirinae, distinguished by the shape of their nucleocapsids [3]. The subfamily Coronavirinae consists of four genera: the alpha-, beta-, gamma-, and delta-coronaviruses. There are seven coronaviruses infecting people: four of them causing a common cold (229E, NL63, OC43, HKU1), and three are associated with potentially severe respiratory symptoms, namely severe syndrome coronavirus (SARS-CoV), Middle East respiratory syndrome coronavirus (MERS-CoV), and the emerging type of SARS-CoV-2, which has 79% sequence homology with SARS-CoV [1]. The factors responsible for unusually high pathogenicity of SARS-CoV and MERS-CoV are not entirely understood. However, research on these pathogens laid the groundwork for quick deciphering of SARS-CoV-2′s life cycle at the beginning of the pandemic. Coronaviruses are the largest known RNA viruses, with genomes ranging from 25 to 32 kb and 118–140 nm in virions diameter. Almost two-thirds of the genome encodes non-structural proteins (nsps), participating in transcription, RNA genome replication, and counter-immune activities. Coronavirus nsps are initially synthesized as long precursor polypeptides, cleaved by virally encoded proteases. Among these nsps, nsp12 is the large RNA-dependent RNA polymerase (RdRp), which in complex with other CoV nsps replicates RNA genome. Another part of the genome encodes structural proteins: spike S protein, for viral entry; envelope E (or HE in torovirus) protein, forming a pivotal ion channel in viral maturation and propagation process; and membrane M protein, for viral structure assembly and binding with nucleocapsid N protein, which binds to the viral RNA itself. The rest of the genome can be translated as accessory proteins that help the virus to evade immunological inhibition [3]. 

SARS-CoV-2′s S protein contacts the host cell through a receptor-binding domain (RBD). RBD recognizes and binds to angiotensin-converting enzyme 2 (ACE2) [4,5,6], and this interaction is facilitated by the presence of neuropilin-1 [7]. Upon ACE2 binding, SARS-CoV-2 utilizes two strategies to get inside of the cell depending on the expression patterns of host cell surface proteinase [8,9]. On the one hand, the S protein is cleaved by cathepsin-L, leading to the virus’s endocytosis. Alternatively, the S protein can also be activated by the transmembrane serine protease TMPRSS2/4, which exposes a hydrophobic pocket and induces quick internalization directly through the plasma membrane. The viral genome can be ejected from the virus directly into the host cytoplasm after the spike protein folds back to itself [6,8,9,10]. Then, the viral genomic RNA can be subsequentially translated by the host ribosome as a messenger RNA (mRNA).

**Figure 1:**
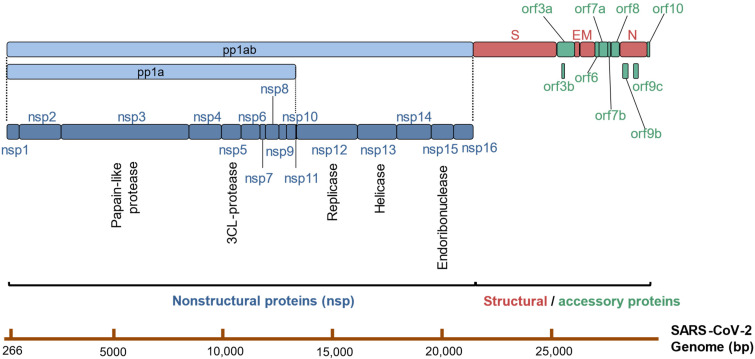
The principal scheme of the SARS-CoV-2 genome. Bars on the bottom represent each known viral protein, and size of each bar is determined by the length of its coding sequence. Dark light bars indicate the coronaviral polyproteins pp1ab and pp1a. Dark blue bars stand for the non-structural proteins, red bars represent structural proteins, and green bars imply accessory proteins.

SARS-CoV-2’s genomic RNA consists of at least 14 open reading frames (orfs) (Figure 1). The first orf accounts for two-thirds of its genome. This giant orf1 is firstly read by host ribosomes encoding two big polyproteins, pp1a and pp1ab. These proteins subsequentially are cut into 16 non-structural proteins (nsp1–11 from pp1a, nsp1–10, and nsp12–16 from pp1ab) by the viral cysteine proteases 3-chymotrypsin–like proteases (3CLpro or M^pro^, nsp5) and papain-like proteases (PLpro, nsp3) [5,11]. After they are released from the polyproteins, nsp2–16 form viral replication and transcription complex (RTC) that is involved in viral RNA synthesis, RNA proofreading, and RNA modification. Like other coronaviruses, SARS-CoV-2′s genomic RNA is synthesized by nsp12 RNA-dependent RNA polymerase (RdRp) with two cofactors, nsp7 and nsp8. The RdRp has been proved to be the centerpiece in the virus life cycle, being critical not only for replication of the viral genome but also for transcription of subgenomic RNAs (sgRNAs) [12]. Some of the nsps (nsp3, nsp4, nsp6) have transmembrane domains to anchor the replication–transcription complex to the cell endomembranes and turn them into viral replication organelles, a prerequisite for the synthesis of additional viral RNAs [13,14]. With all that being said, the translation products of orf1 are extremely critical for the virus to survive in the host cells, which could be one of the reasons that orf1 constitutes the majority of the virus genome.

To succeed in the host cells, the virus must replicate itself as much as possible before it gets eliminated by the host immunity or loses genome integrity due to its mutations. In the pathologic organelle, termed ERGIC (endoplasmic reticulum-to-Golgi intermediate compartments), the four essential structural proteins (S, E, M, N) together with the virus genomic ssRNA are assembled into complete virions, and are subsequently encapsulated and released from the host cells for another round of infection in the host body [15]. The nascent viruses are exhaled from the nasal passages of the patients and spread among the population.

Fortunately, the human body developed multiple strategies during evolution to counter viral infection. Interferon response is the main antiviral defense in almost all cells in the body, which is modulated by pattern-recognition receptors (PRRs).

## 3. Pattern-Recognition Receptors and Interferons

### 3.1. Pattern-Recognition Receptors

Pattern recognition receptors (PRRs) are intracellular molecular sensors recognizing abnormal and pathogen-associated molecular patterns (PAMPs), such as double-stranded RNA, unmethylated DNA, and bacterial or viral fragments [16]. Since PAMPs are unique and highly conserved molecular structures associated with the specific kind of pathogenic microorganisms, the host body evolutionally developed a set of PRR sensors and gene-responders, defined as the innate immune system [17,18,19,20]. Not only viral infection, but the host cell itself can also produce some proteins and metabolites that PRRs could recognize, notably aberrant transcripts or proteins, as well as the products of cell necrosis and tissue damage. Such host molecules were defined as “damage-associated molecular patterns” (DAMPs) [19,20,21]. PPRs are expressed in immune cells, including monocytes, neutrophils, macrophages, dendritic cells, natural killer (NK) cells, mast cells, eosinophils, and basophils [22], and non-immune cells such as fibroblasts and epithelium cells, including almost all kinds of epithelial cell types, notably oral, pharyngeal, esophageal, intestinal, cervical, kidney, and airway epithelial cells [23,24,25,26]. PPRs can be found throughout the airway epithelium, from nasal tunnel to alveoli [27,28,29]. PPRs can directly recognize specific PAMPs or DAMPs on the surface of cells, in engulfed endosomes, or in the cellular milieu. This allows host cells to distinguish “self/ healthy” and “non-self/unhealthy” [30,31,32]. After recognition and relevant ligand binding, PRRs can initiate nonspecific innate immune activities, such as activating specific genes (notably IFNs), expressing and secreting specific sets of cytokines, and facilitating cell apoptosis or pyroptosis, to induce inflammation and delay the spread of pathogens. The second important function of PPRs is to activate adaptive immune response and modulate its activity [33]. 

Based on their protein domain homology, most PRRs in the innate immune system can fall into several categories: toll-like receptors (TLRs), retinoic acid-inducible gene-I (RIG-I)-like receptors (RLRs), nucleotide oligomerization domain (NOD)-like receptors (NLRs), C-type lectin receptors (CLRs), absent in melanoma-2 (AIM2)-like receptors (ALRs), and cytosolic nucleic acid sensor cyclic GMP- AMP (cGAMP) synthase (cGAS) [24,34]. Among them, TLRs, RLRs, and cGAS are the primary virus sensing receptors, which we will focus on in this review.

TLRs are highly conserved proteins from the worm to mammals [17,35,36,37,38,39]. They are type I integral membrane glycoproteins, consisting of the extracellular domains containing several leucine-rich-repeat motifs that recognize PAMPs or DAMPs, the transmembrane domains, and a cytosolic signaling TIR domain that is responsible for downstream signal transduction [40,41,42,43,44,45]. There are 13 TLRs (TLR1–13), including 10 functional TLRs (TLR1–10) in humans and 12 TLRs (TLR1–9, TLR11–13) expressed in mice [24]. To sense different PAMPs, TLRs have various expression patterns. Some TLRs (TLR1, 2, 4, 5, 6, 11) are expressed on the cell membranes in the form of heterodimers (TLR1/2, TLR2/6) or homodimers (TLR4, 5, 11), recognizing mainly bacterial lipids, lipoproteins, and lipopolysaccharides (LPS). Another set of TLRs (3, 7, 8, 9) is mainly expressed in subcellular organelles such as the endoplasmic reticulum (ER), endosomes, lysosomes, and endo-lysosomes; they recognize aberrant nucleic acids from viruses and other microorganisms. Among these intracellular TLRs, TLR3 recognizes viral double-stranded RNA (dsRNA) metabolites in vesicles as well as those on the cell surface, while TLR7 and TLR8 can recognize single-stranded RNA (ssRNA) inside of vesicles, chasing after viral infection. TLR9 is a vesicular sensor for viral DNA and host DNA leakage [23]. Activated TLRs induce downstream IFN-I and NF-kB signaling pathways, resulting in the expression of proinflammatory cytokines, attraction to immune cells, and activation of type I and type III interferons signaling (see details in Type I Interferon Pathway) [46].

RLRs are the family of intracellular PRRs which recognize viral RNA in the cytoplasm [47]. The RLR family includes RIG-I, melanoma differentiation-associated gene 5 (MDA5), laboratory of genetics and physiology 2 (LGP2). RLRs’ function can be interpreted from the structure standpoint. In the N-terminus of RIG-1 and MDA5, two caspase activation and recruitment domains (CRAD) are responsible for transmitting signals to downstream interactors [48,49]. The repressor domain (RD) at the end of the C-terminus of RIG-I and LGP2 inhibits the activation of the receptor [50,51]. However, MDA5 does not have self-inhibitory function because it lacks repressor domain [49,52]. All three RLRs share the middle DexD/H helicase domain and the C-terminal domain (CTD). The DexD/H helicase domain functions as ATPase helicase, while the CTD activates the protein itself with presence of viral RNA [53,54]. RIG-I can recognize dsRNA shorter than 1000 bp and 5′-triphosphate RNA of viruses, while MDA5 recognizes long-chain dsRNA that is greater than 1000 bp [55,56]. It is reported that LGP2 assists MDA5-RNA interactions, which leads to enhanced MDA5-mediated antiviral signaling by regulating MDA5. LGP2 increases the initial rate of MDA5-RNA interaction and regulates MDA5 filament assembly [57]. For SARS-CoV-2, RLRs should be a severe threat since both RIG-I and MDA5 are very effective against a few RNA and some DNA viruses.

cGAS is a cytosolic double-stranded DNA (dsDNA) sensor that activates interferon responses through the production of the second messenger cGAMP that activates the adaptor stimulator of interferon genes (STING), which is the so-called cGAS-cGAMP-STING pathway [34]. In humans and other mammals, independently of the DNA sequences, cGAS binds to the sugar-phosphate backbone of dsDNA coming from bacteria, DNA viruses, retrovirus, dead cells, and even self-DNA, which induces the conformation change of cGAS in its active site. Activated cGAS catalyzes 2′3′-cGAMP, which functions as a second messenger that binds to STING and leads to the translocation of STING from the ER to the ERGIC. STING is believed to induce the phosphorylation of TBK1, IKK, and IRF3 after translocation and therefore to induce interferon responses and signaling [34,58,59,60]. In other animals such as Drosophila, it is a different story. Recently, it was reported that cGAS-like receptors (cGLRs) are nucleic acid receptors in Drosophila, which catalyze 3′2′-cGAMP and activate Sting-dependent antiviral responses [61,62].

### 3.2. Type I Interferon Pathway

Activation of any nucleic acid PRR sensor results in universal antiviral immune responses, with an essential role in type I and III interferon signaling. Type I interferon, or IFN-I, is the family of related genes among the first responders to viral infections. In humans, there are five major groups within the type I interferon family, designated IFN-α, β, κ, ε, and ω. Since virtually every cell in the body can produce IFNs, their expression has to be tightly controlled, and it is typically not secreted unless the cell becomes infected or encounters other types of stressors [23]. As discussed above, PRRs can be broadly divided into two groups. Sensors from the first group are expressed in the cytoplasm, consisting of nucleic acid sensors (NA sensors), which recognize immunogenic RNA (RIG-I, MDA5) or DNA molecules (cGAS, STING), and bacterial peptidoglycans sensors termed NOD1 and NOD2. The second group of sensors is the TLRs and CLRs, which are mainly expressed on the membranes. Activation of any of these receptors induces activation of key signaling kinase complexes TBK1/IKKε and IKK1/IKK2. While all membrane-bound receptors require adaptor proteins MyD88 or TRIF to activate downstream signaling kinases, cytosolic NA sensors (RIG-I, MDA5, cGAS, STING) activate TBK1/IKKε directly. Activated TBK1/IKKε complex phosphorylates members of IRF transcription factors family, while IKK1/IKK2 complex controls activation of NFκB transcription factors. For the antiviral products, NFκB regulates the expression of inflammatory cytokines, while IRF transcription factors are the key regulators of the type I IFN response leading to secretion of IFNs [63]. 

The type I IFN molecules activate cells in both autocrine and paracrine manner and in a self-promoting way. Secreted IFNs are recognized by the interferon-α receptor complex (IFNAR) consisting of two transmembrane proteins, IFNAR1 and R2. Activation of IFNAR mediates downstream predominantly through the JAK/STAT signaling, which promotes further activation of type I IFN response, as well as inflammatory responses through the NFκB pathway. Moreover, the type I IFN is closely linked to the cell apoptosis: under certain circumstances such as viral infection, activation of the interferon-dependent pathways can promote apoptosis or pyroptosis of the cell. Molecules such as receptor-interacting protein kinase 1 (RIPK1), RIPK3, FAS-associated death domain protein (FADD), FLICE-like inhibitory protein (FLIP), and several caspases, which are essential regulators of different forms of cell death, are incorporated into signaling of TLRs, NOD-like receptor, and NA sensors [64]. These signaling modules have high capability to switch the cell status from inflammation to cell death, which is a core feature of IFN protection against viral infections.

The interferon system provides effective protection from coronaviruses. Recent COVID-19 clinical data have shown that the IFN response in epithelial cells of the upper respiratory tract can curb SARS-CoV-2 replication. Some risk factors, for example, delayed or decreased interferon signaling [65], neutralizing of IFN-I by autoantibody [66], and inappropriate inflammatory cytokine responses, may favor developing more severe COVID-19 symptoms [67]. Interestingly, such correlation is always shifted during early infections when the virus is still active [68]. Accordingly, SARS-CoV-2 viral proteins directly or indirectly attempt to inhibit IFN-I response to gain extra time for safe replication. Trying to outsmart each other, host and virus show the typical pattern of the evolutional arms race when both partners engage multiple intelligent molecular tools in order to survive.

## 4. Host-Virus Arms Race

### 4.1. Arms Race in Pathogen Sensing and IFN Induction

**Figure 2:**
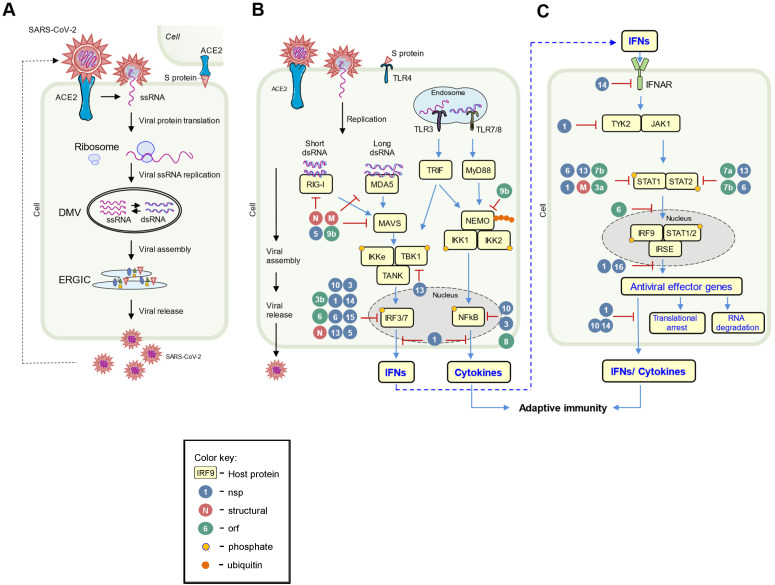
The replication cycle of SARS-CoV-2 and viral-host interplay. (**A**) SARS-CoV-2 enters the cell via ACE2. Viral RNA gets translated by host ribosomes and replicated within the double-membrane vesicles. The virions are assembled at ERGIC and then egressed outside the cell. (**B**) Host cell detect viral proteins and nucleic acids through PRRs and induce interferon pathway signaling. (**C**) Activated interferon response through JAK/STAT pathway inhibits viral cycle unless blocked by viral proteins. Yellow squares represent host proteins participating in antiviral response; orange dots illustrate phosphorylation events. Red circles stand for coronaviral structural proteins; green for orfs; and blue for nsps.

Like other viruses, SARS-CoV-2 produces a few PAMPs, including its ssRNA, dsRNA, S protein, and E protein, which the PPRs of innate immune system can directly recognize. Human cells can detect SARS-CoV-2 virus through several mechanisms (Figure 2). After injection or endocytosis, SARS-CoV-2′s genomic ssRNA can be recognized by endosomal TLR7 and TLR8 [9,69]; its intermediate dsRNA generated during viral replication is sensed by endosomal TLR3 and cytosolic RIG-I and MDA5 [70]; and viral S protein may activate TLR4 [71,72], while E protein directly triggers TLR2 downstream signaling [73]. Upon PAMPs recognition, membranous PRRs activate downstream signaling through specific adaptor proteins (MyD88 for all TLRs; TRIF for TLR3; IRIF, TRAM for TIR4) to active NF-κB, MAPK, and IFN-I pathways. Correspondingly, activated transcription factors (RelA, IRF3/7, AP-1) induce expression and secretion of proinflammatory cytokines and chemokines (such as IL-6, TNF pro–IL-1β, and IL-8), type I (mostly α and β) and type III (γ1/2/3) IFNs [46,74]. In the cytoplasm, the presence of viral RNA metabolites should be recognized by RIG-I and MDA5, which activates IFN-I response through signaling kinases IKKε/TBK1 [70] and cytokines released through the NFκB pathway. 

Besides PAMPs recognition, direct dsRNA-induced activation through RNA-activated protein kinase R (PKR) and 2′-5′-oligoadenylate synthetase-like (OASL) was observed in infected SARS-CoV-2 respiratory epithelial-derived cells and cardiomyocytes [75]. In addition to the rapid interferon and inflammatory response against the PAMPs, the viral infection could lead to host cell apoptosis followed by releasing self-DNA from the nucleus into the cytoplasm, which is one of DAMPs and can directly trigger cGAS/STING and AIM2/IFI16 pathways that induce more interferon responses [70]. The innate immune system can activate the adaptive immune response upon recognition of pathogens, including activation of T cells and B cells [33]. Therefore, the virus has very little space to succeed if innate immunity is fully active.

However, SARS-CoV-2 is quite smart and can directly inhibit multiple pathways of innate immune responses at all stages of infection. In addition to their essential roles in the viral life cycle, most SARS-CoV-2 viral proteins antagonize core cellular functions in human cells to evade host immune responses in favor of the virus (comprehensive summary, see Figure 2 and Table 1). In the next few paragraphs, we describe antagonism by SARS-CoV-2 to avoid host antiviral responses.

Starting with the nsps, nsp3, the papain-like protease, preferentially cleaves the ubiquitin-like interferon-stimulated gene 15 protein (ISG15) from interferon responsive factor 3 (IRF3), and thereby inhibits type I interferon responses [87]. It is also reported that nsp3 can strongly impair the activity of IFNA4 and IFNB1, IRF3 binding, and NF-kB binding [88].

Nsp5 inhibits dsRNA-induced IFNs induction from RIG-I–mitochondrial antiviral signaling (MAVS) protein–IFN pathway by cutting off RIG-I’s 1–10 amino acids and suppressing its activation MAVS, also increasing the ubiquitination level of MAVS and facilitating proteasomal degradation of MAVS [90]. Furthermore, nsp5 was reported to inhibit INF-β transcription induced by TBK1 and IKKε via blocking the nuclear translocation of phosphorylated IRF3 [92]. Nsp6 suppresses the phosphorylation of IRF3 by binding and inhibiting TBK1 phosphorylation [74]. 

Another non-structural protein, nsp10, was reported to impair the activity of type I interferons by interacting with IRF3 and NF-kB signaling and suppressing cytokines production upon infection [88]. This activity is probably mediated by the nsp10′s RNA 2-0-methylation activity, known as immunosuppressive RNA modification, that allows the virus to attenuate RIG-I/MDA-5 recognition [109,110]. 

Interestingly, nsp12 was reported attenuating type I interferon production by inhibiting IRF3 nuclear translocation [111], not like any of its homologs, whereas other data suggested otherwise [112]. The nsp13 protein is believed to interact with signaling kinase TBK1 and block its activation [79,83]. Moreover, it is reported to downregulate primary interferon production by limiting the nuclear localization of IRF3 [93]. Furthermore, nsp13 is involved in viral RNA 5′ cap synthesis: a modification that must be present on the 5′ end of every mRNA, helping the virus to avoid recognition by RIG-I [113].

Nsp15 was reported to inhibit the nuclear localization of IRF3, and therefore suppressing interferon induction and signaling [88]. It is believed that nsp16 together with nsp10 can modify the cap of viral RNA by adding a methyl group at the 2′-O position of the first nucleotide, which prevents RIG-I detection [76,114]. 

Not only can non-structural proteins play against PRR sensors, but the structural M protein interacts with the central adaptor protein MAVS in the innate immune response pathways, impairing MAVS aggregation and its recruitment of downstream TRAF3, TBK1, as well as the IRF3 transcription factor, which results in impaired antiviral response [98]. Another study reported that SARS-CoV-2′s M protein also suppresses type I and III IFN expression by targeting RIG-I/MDA-5 signaling to attenuate antiviral immunity and enhance viral replication [99]. The N protein of SARS-CoV-2 inhibits TRIM25/RIG-I interaction through binding to the RIG-I protein’s DExD/H domain. The N protein also impairs TBK1/IRF3 association, preventing nuclear translocation of IRF3 and IFNs expression [100,101]. 

Many accessory proteins were also reported to inhibit interferon induction. Orf3b functions as a potent IFN antagonist by preventing the nuclear translocation of IRF3, of which the suppression of IFN induction depends on the length its C terminus [102]. Orf6 localizes at the nuclear pore complex and directly interacts with Nup98-Rae1 through its C-terminal domain to impair the docking process of cargo-receptor (karyopherin/importin) complex, and thereby blocks STAT1, IRF3, and ISGF3 nuclear translocation [83,93,103,104]. Orf9b accumulates immediately after release during SARS-CoV-2 infection and inhibits the RIG-I/MAVS pathway-dependent type I interferon response by interrupting the K63-linked polyubiquitination of the interferon signaling modulator NEMO [107]. Orf9b was also reported to interact with RIG-I, MDA-5, MAVS, TRIF, STING, and TBK1 and impede the phosphorylation and nuclear translocation of IRF3 [108]. 

Notably, after infection, the virus generates double-membrane vesicles (DMVs) as its viral replication organelles to physically separate viral biochemistry from cellular cytoplasm, in this way avoiding PRRs recognition [115]. 

### 4.2. Arms Race in IFN Signaling

Nsp1 is also able to inhibit IFN signaling in part by blocking STAT1, IRF3 phosphorylation, Tyk2, and STAT2 activation [83,84]. It was reported that the 500–532 deletion in the nsp1 coding region led to lower IFN-I response, which suggests nsp1 is closely associated with immune counteraction [85]. Nsp3′s macrodomain can also reverse PARP9/DTX3L-dependent downstream ADP-ribosylation, induced by interferon signaling [89]. Nsp5 was reported to inhibit INF-β transcription induced by TBK1 and IKKε via blocking the nuclear translocation of phosphorylated IRF3 and impairing type I IFN signaling by inducing phospho-STAT1/2 accumulation [88,91]. Nsp6 impairs phosphorylation of STAT1 and STAT2 [83]. Nsp8 and nsp9 interact with 7SL RNA component of SRP54 and SRP19, the components of the signal recognition particle (SRP), which recognizes the signal peptide of secretory proteins. By disrupting protein trafficking, these two nsps affect cytokine secretion and MHC-I recycling and induce significant reduction of the immune response [76]. The nsp13 protein block the phosphorylation of transcription factor STAT1 [116]. Nsp14 inhibits IFNs signaling differently by facilitating lysosomal degradation of the essential interferon receptor IFNAR1. A lower number of IFN receptors slows down autocrine and paracrine activation of STATs transcription factors and ultimately results to weaker immunity [88,93]. 

Two accessory proteins, orf7a suppresses the IFN-I response by inhibiting STAT2 phosphorylation, while orf7b inhibits the phosphorylation of both STAT1 and STAT2 [83]. Orf8 can strongly inhibit type I interferon (IFN-β) and NF-κB-responsive promoter, as well as the interferon-stimulated response element (ISRE) [104,106]. 

### 4.3. Arms Race between Virus and IFN-Induced Effectors, and Cellular Events

Right after it is released from the pp1a and pp1ab, nsp1 binds to 18S ribosomal RNA and 40S subunit at the mRNA entry channel of the ribosome, which results in global inhibition of mRNA translation upon viral infection, to earn extra time for the virus to replicate itself before the cell can shut down the translation machinery or induce self-degradation [76,77,78,79,86,117,118]. Not only that, nsp1 blocks nuclear export of host mRNAs, including IFNs, by preventing the proper binding of NXF1 to mRNA export adaptors and NXF1 docking at the nuclear pore complex [81,82], and hypothetically through enigmatic interaction with the primosomal proteins POLA1, POLA2, PRIM1, and PRIM2 [79,80]. Nsp14 in complex with nsp10 can also distort host translation processes due to their exonuclease and N7-MTase activities [94]. Nsp16 binds to the pre-mRNA recognition domains of the U1 and U2 splicing RNAs and therefore suppresses host mRNA splicing during SARS-CoV-2 infection [76]. In this way, the expression of interferon-induced genes is greatly attenuated.

Besides direct molecular confrontations, SARS-CoV-2 also affects macromolecular processes, modulating cell autophagy, fusion, and programed cell death. Autophagy plays an important role in regulating immunity-related cell death and antiviral responses. Similar to blunting PRR signaling, SARS-CoV-2 is also attempting to distort autophagy. The nsp15 protein inhibits de novo autophagy induction [88], while the E and M structural proteins were reported to block autophagic turnover in the host cells to prevent degradation of assembled virions [88]. Autophagy is also modulated by SARS-CoV-2 accessory proteins, e.g., orf3a inhibits autophagic turnover by targeting the late endosomes and blocking its fusion with autophagosomes [88]. Another protein orf7a was reported to block autophagic turnover by affecting lysosomal acidification [88]. The orf8 protein directly interacts with MHC-Ι molecules and facilitates their lysosomal degradation via autophagy, therefore, SARS-CoV-2–infected cells are much less sensitive to be lysed by cytotoxic T lymphocytes [105]. 

Finally, SARS-CoV-2 could induce direct cell fusion, which is another mechanism for viral spreading. During the virus assembly process, the S protein with other structural proteins (E, M, N) are translocated into the lumen of the intermediate compartment at the endoplasmic reticulum (ER)-Golgi interface [119]. Then, the matured virions traffic to de-acidified lysosomes and egress by Arl8b-dependent lysosomal exocytosis to start another round of infection [120]. However, the S protein also translocates to the surface of infected cells through the COPI (retrograde) and COPII (anterograde) transport, leading to increased TMEM16F expression, which in turn increases the phosphatidylserine concentration on the plasma membrane. The interaction between the S protein on the infected cell surface with the ACE2 receptor on the neighboring cell and the increased concentration of phosphatidylserine may induce syncytia formation. In this manner, SARS-CoV-2 could spread through the tissue remaining inside the cells, out of reach of adaptive immunity [95,96,97]. Notably, in terms of the arms race, the evolution of the S protein contributes to the escape from the adaptive immune system, helping the virus to extend replication time [121]. Indeed, other than the S protein, it was reported that the Alpha (B.1.1.7) variant has dramatically increased protein levels of N, orf9b, orf6, and sgRNAs, which makes it more effectively dampen down epithelial cell innate immune responses in the airway [122].

## 5. Beyond the Nature—Antiviral Pharmaceuticals

**Figure 3:**
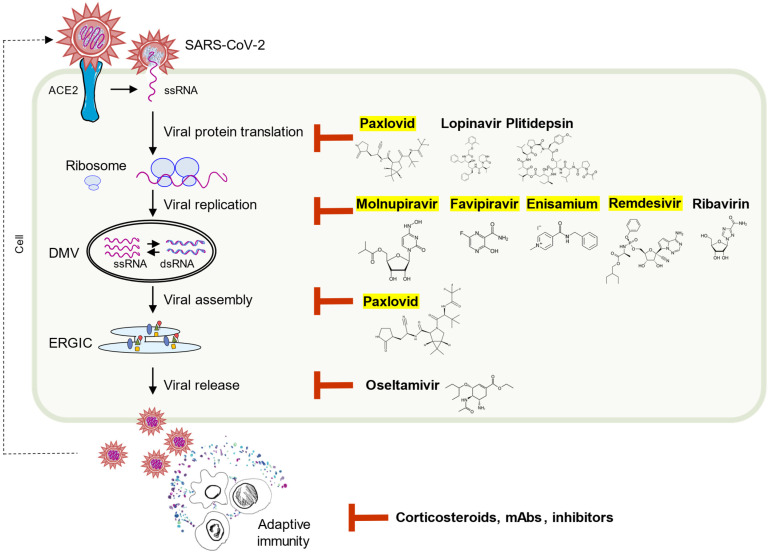
Therapeutics used against SARS-CoV-2 infection. Pharmaceuticals that can potentially inhibit the replication stages of RNA virus during its infection are labeled with their molecular formula. Red bar-headed lines indicate the inhibition effect of the drug. Anti-COVID-19 drugs are highlighted in yellow.

Such complicated viral counterintelligence aims to blunt host’s innate immunity and earn extra time for its replication. However, humans have one more weapon: pharmaceuticals. The viral protease and RNA-dependent RNA polymerase (RdRp) have been proven to be the bottleneck of many viruses. Viruses generate a variety of aberrant nucleic acids during their distinctive replication cycles. Any delay in the viral replication process generates a variety of uncut polyproteins and not finished viral genomic RNA polymers, which are the main targets that can be recognized by relevant PRRs, leading to activation of type I/III interferon signaling [12]. Therefore, it is not surprising that the three most effective anti-COVID clinical drugs target viral replication and translation processes (Figure 3).

The well-known drug Remdesivir is a nucleotide prodrug of an adenosine analog, initially developed for treatment of the Ebola virus. Remdesivir binds to the viral RdRp and suppresses viral replication by prematurely terminating RNA transcription. It is reported that Remdesivir is able to act against SARS-CoV-2 in vitro [123]. Additionally, Remdesivir treatment can be quickly initiated after inoculation in a SARS-CoV-2 infection rhesus macaque model and compared with the control group, those animals who received remdesivir had lower virus loads in the lungs as well as milder lung damage [124]. Furthermore, many clinical trials showed that Remdesivir benefits the patients with mild to moderate COVID-19 at the early stage [125,126,127,128,129]. 

Molnupiravir is an oral prodrug of beta-D-N4-hydroxycytidine (NHC) developed by Merck. It functions as a ribonucleoside that has broad antiviral activity towards RNA viruses. NHC uptake by viral RdRp can lead to a great number of viral mutations and some of them are very likely lethal mutagenesis [130]. Molnupiravir is approved by the Food and Drug Administration (FDA) for emergency use for adult COVID-19 patients within 5 days of symptoms onset [131,132,133,134]. However, it is also reported that Molnupiravir could introduce mutations into the viral genome and the mammalian cells [135]. Therefore, the FDA suggested that Molnupiravir can only be administrated for 5 days when other options, such as Remdesivir or Nirmatrelvir (Paxlovid), are not appropriate for the patients.

Nirmatrelvir is also an oral drug against COVID-19, developed by Pfizer. Unlike Remdesivir and Molnupiriavir, Nirmatrelvir is a protease inhibitor targeting the Mpro of all coronaviruses known to infect humans [136,137]. The M^pro^, or 3CLpro, is one of the viral proteases that cleaves the two orf1 polyproteins, and thereby plays a critical role during viral replication (See details in SARS-CoV-2 Life Cycle). Based on data from clinical trials, the FDA approved the use of Nirmatrelvir combined with Ritonavir (boosting agent) in patients older than 12 years within 5 days of symptom onset [138,139].

SARS-CoV-2 keeps mutating itself to avoid immune clearance, which raises concern that those drugs might lose function in the patients infected with variants. For instance, the most notable variants of concern, named respectively as alpha/B.1.1.7 (multiple mutations in S protein, N protein, orf1ab, and orf8), beta/B.1.351 (mutations in S protein and nsp6), gamma/P.1(mutations in spike protein and nsp6), delta/B.1.617.2 (multiple unexpected mutations in S protein), and Omicron/ B.1.1.529 (at least 32 mutations in S protein, which include those mutations of other variants, i.e., alpha, beta, gamma, and delta), are more transmissible and likely more deadly because their increasing ability to bind host ACE2 receptors and to evade immune clearance, except the Omicron variant [140,141,142,143,144,145]. The Omicron variant seems to be more contagious, which makes sense because the high number of mutations in its spike protein, but surprisingly less fatal [146]. However, it is believed that the protease inhibitors––Remdesivir, Molnupiravir, and Nirmatrelvir––stay active against the five variants of concern mentioned above [147].

Even those drugs with weak pharmaceutical activity toward SARS-CoV-2 work well at the early stages of infection when multiplication of viruses occurs. For example, Enisamium iodide, an isonicotinic acid derivative, which was reported as an inhibitor of RdRp of influenza A and B virus strains, recently was suggested to have the potential to prevent severe development of COVID-19 by inhibiting RNA synthesis of SARS-CoV-2 [148,149,150,151,152]. Moreover, Enisamium can directly inhibit influenza and SARS-CoV2 RNA replication, and recent clinical trials showed that COVID-19 patients recover faster and safer when prescribed with Enisamium compared to the placebo group [152]. Meantime, the popular antiviral composition Lopinavir/Ritonavir (HIV 3CLpro inhibitors) and other HIV protease inhibitors did not reveal any efficacy to improve the outcomes of COVID-19 patients based on multiple studies [153,154,155].

Indeed, multiple clinical trials showed that inhibitors of RdRp and viral proteases, taken during the first 3–4 days of infection, dramatically improved the chances of patients to avoid a severe or prolonged COVID-19 course. Given that these inhibitors impair RdRp or protease activity, they can increase the concentration of abnormal viral RNA and proteins to the levels visible for inhibited PRR/IFN-I pathways. This could facilitate immune clearance and even pre-activate cells before invasion. Altogether, antiviral drugs seem to help PRRs detect infectious agents, while the well-timed interferon response appears to be the key factor preventing severe development of the COVID-19. However, the arms race is underway: once a new drug is released, the virus will rapidly start another round of evolution. Therefore, innate immunity and its multiple antiviral sensors remain humans’ main ally in the pandemic world.

## 6. Conclusions and Future Outlook

Timely and robust innate immune responses in the airways through activation of PRR-Interferon signaling have been confirmed by numerous studies to be a critical factor to restrict SARS-CoV-2 infection, viral replication, and controlling the disease course. There are many fundamental differences between adults and children, which help us understand why kids are less likely to develop severe illness from SARS-CoV-2 [156]. For example, the expression of ACE2 receptor is upregulated in males and the elderly compared to children, which enables increased virus entry to the cells [157]. Moreover, kids have less chance to have severe comorbidities, such as metabolic or cardiovascular diseases. Older or susceptible people, especially those with systematic conditions such as obesity, diabetes, or hypertension [158,159,160], often possess compromised innate immunity and defective early interferon response, which is linked to higher viral load, longer infections, and multi organ failure [156,161]. Hence, understanding the complex interplay between host immunity and SARS-CoV-2 is critical to determine the most appropriate approach for an individual patient at a given time.

This review provides a systematic overview of the biological basis of the SARS-CoV-2 life cycle and the immune arms race between host and virus. The innate immune response starting with PRRs recognitions, interferon induction, and signaling is believed to be the first line to fight against SARS-CoV-2 infection. We also review a few antiviral pharmaceuticals—mainly protease inhibitors—which seem to have promising results in clinical trials. It is suggested that applying those antiviral drugs at the early stages of COVID-19 (within 5 days) is the most effective strategy to manage this disease.

Still, only few drugs are available for COVID-19 treatment compared to the dramatically increasing number of patients. More detailed studies on the molecular biology of SARS-CoV-2 and the discovery of both new and repurposed drugs should be the focus of the future.

## Figures and Tables

**Table 1:** Immunosuppressor activity of SARS-CoV-2 proteins.

SARS-CoV-2 Proteins	Target	Mechanism	References
Nsp1	STAT1; STAT2; IRF3; Tyk2; 18S rRNA; 40S ribosomal and primosomal subunit	Translation inhibition by interfering with nuclear export of host mRNA; blocks IRF3 nuclear translocation; inhibition of STAT1 phosphorylation; reduced expression of STAT2 and Tyk2	[76,77,78,79,80,81,82,83,84,85,86]
Nsp3	NF-kB; ISG15; IRF3; PARP9/DTX3L	Cleaves ISG15 from IRF3; inhibits IFN-I promoters, IRF3 and NF-kB binding sites; reverses PARP9/DTX3L-dependent ADP-ribosylation	[87,88,89]
Nsp5	IRF3; STAT1; STAT2; RIG-I; MAVS	Cleaves off the 10 most-N-terminal amino acids from RIG-I; promotes the ubiquitination and proteosome-mediated degradation of MAVS; inhibits blocking the nucleus translocation of phosphorylated IRF3; induces phospho-STAT1/2 accumulation impairing type I IFN signaling	[88,90,91,92]
Nsp6	STAT1; STAT2; IRF3; TBK1	Suppresses phosphorylation of IRF3 by binding to TBK1; inhibits STAT1 and STAT2 phosphorylation	[83]
Nsp8	7SL RNA Component of SRP54	disrupts protein trafficking for secretion or membrane integration	[76]
Nsp9	7SL RNA Component of SRP19	disrupts protein trafficking for secretion or membrane integration	[76]
Nsp10	IRF3; NF-kB	Impairs the activity of IFNA4 and IFNB1, IRF3 binding and NF-kB binding, and suppresses cytokines production	[88]
Nsp13	STAT1; STAT2; TBK1; IRF3	Inhibits TBK-1, STAT1 and STAT2 phosphorylation; blocks IRF3 nuclear translocation	[83,88,93]
Nsp14	IRF3; IFNAR1	Inhibits IRF3 nuclear translocation; induces lysosomal degradation of IFNAR1; inhibits host cellular translation via ExoN and N7-MTase activities	[88,93,94]
Nsp15	IRF3; early autophagosome	Inhibits IRF3 nuclear translocation; inhibits de novo autophagy induction	[88]
Nsp16	U1 and U2 splicing RNAs	Suppresses host mRNA splicing through binding to the pre-mRNA recognition domains of the U1 and U2 splicing RNAs	[76]
S	ACE2	Evades host cells and induces syncytia formation through binding to ACE2 receptor	[95,96,97]
E	Autophagosome	Blocks autophagic turnover	[88]
M	RIG-I; MDA-5; MAVS; autophagosome	Impairs MAVS aggregation and recruitment of downstream components; induces LC3B accumulation in the perinuclear space; suppresses type I and III IFN expression by targeting RIG-I/MDA-5 signaling	[88,98,99]
N	TBK1; IRF3; RIG-I	Binds to with the RIG-I protein at its DExD/H domain and suppresses IFN-β production; impairs TBK1/IRF3 association and IRF3 nuclear translocation	[100,101]
Orf3a	STAT1; lysosomes; autophagosomes	Inhibits STAT1 phosphorylation, blocking the fusion of lysosomes with autophagosomes	[83,88]
Orf3b	IRF3	Inhibits IRF3 nuclear translocation	[102]
Orf6	IRF3; STAT1; KPNA2; ISGF3	Interacts with KPNA2; blocks STAT1, IRF3 and ISGF3 nuclear translocation	[83,93,103,104]
Orf7a	STAT2; lysosomes	Inhibits STAT2 phosphorylation; decreases lysosomes acidification	[83,88]
Orf7b	STAT1; STAT2	Inhibits STAT1 and STAT2 phosphorylation	[83]
Orf8	NF-κB; MHC-Ι molecules	Mediates their lysosomal degradation of MHC-I molecules; inhibits NF-κB-responsive promoter	[93,105,106]
Orf9b	RIG-I; MDA-5; MAVS; NEMO; TRIF; TBK1; IRF3; STING	Interrupts K63-linked polyubiquitination of NEMO and inhibits IFN signaling; interacts with RIG-I, MDA-5, MAVS, TRIF, STING, and TBK1 and impedes the phosphorylation and nuclear translocation of IRF3	[107,108]

## Data Availability

Not applicable.
